# Changes in ontogenetic patterns facilitate diversification in skull shape of Australian agamid lizards

**DOI:** 10.1186/s12862-018-1335-6

**Published:** 2019-01-08

**Authors:** Jaimi A. Gray, Emma Sherratt, Mark N. Hutchinson, Marc E. H. Jones

**Affiliations:** 1 School of Biological Sciences, University of Adelaide, Room 205E, Darling Building North Terrace, Adelaide, SA 5005 Australia; 2 South Australian Museum, Adelaide, SA 5000 Australia; 3 Earth Sciences, Natural History Museum, London, SW7 5BD UK

**Keywords:** Agamidae, Evolutionary development, Geometric morphometrics, Lizards, Ontogeny, Skull

## Abstract

**Background:**

Morphological diversity among closely related animals can be the result of differing growth patterns. The Australian radiation of agamid lizards (Amphibolurinae) exhibits great ecological and morphological diversity, which they have achieved on a continent-wide scale, in a relatively short period of time (30 million years). Amphibolurines therefore make an ideal study group for examining ontogenetic allometry. We used two-dimensional landmark-based geometric morphometric methods to characterise the postnatal growth patterns in cranial shape of 18 species of amphibolurine lizards and investigate the associations between cranial morphology, and life habit and phylogeny.

**Results:**

For most amphibolurine species, juveniles share a similar cranial phenotype, but by adulthood crania are more disparate in shape and occupy different sub-spaces of the total shape space. To achieve this disparity, crania do not follow a common post-natal growth pattern; there are differences among species in both the direction and magnitude of change in morphospace. We found that these growth patterns among the amphibolurines are significantly associated with ecological life habits. The clade *Ctenophorus* includes species that undergo small magnitudes of shape change during growth. They have dorsoventrally deep, blunt-snouted skulls (associated with terrestrial lifestyles), and also dorsoventrally shallow skulls (associated with saxicolous lifestyles). The sister clade to *Ctenophorus*, which includes the bearded dragon (*Pogona*), frill-neck lizard (*Chlamydosaurus*), and long-nosed dragon (*Gowidon*), exhibit broad and robust post-orbital regions and differing snout lengths (mainly associated with scansorial lifestyles).

**Conclusions:**

Australian agamids show great variability in the timing of development and divergence of growth trajectories which results in a diversity of adult cranial shapes. Phylogenetic signal in cranial morphology appears to be largely overwritten by signals that reflect life habit. This knowledge about growth patterns and skull shape diversity in agamid lizards will be valuable for placing phylogenetic, functional and ecological studies in a morphological context.

**Electronic supplementary material:**

The online version of this article (10.1186/s12862-018-1335-6) contains supplementary material, which is available to authorized users.

## Background

The role of natural selection in producing morphological variation is limited by the changes that can be generated by organism-specific processes of growth and development (ontogeny) [1–4]. In each generation, the range of possible forms that natural selection can act upon is limited by the changes that can be generated by growth and development [3, 4]. Throughout their development, organisms can undergo changes in shape, due to differences in relative growth of components, and alterations in timing of their growth, a concept defined as ontogenetic allometry [3, 4]. Studies on ontogenetic allometry have been carried out since 1930, and considerable advances in methods have allowed exploration of patterns in more refined detail [4–10]. These studies have shown that changes in the attributes of ontogenetic patterns are important for facilitating evolutionary processes, [4, 7, 11–13]; evolutionary flexibility of ontogenies has been reported in several recent works e.g. [14–17].

There are two ways in which changes to an ancestral post-natal growth pathway can generate morphological diversity. Firstly, changes in adult shape can occur simply by changing adult size, without altering the shape-size relationship. Modifications in timing or rate of the ancestral growth pathway (heterochrony) account for diversification of shape. Such heterochronic changes, “when a descendant retains the ancestral relationship between size and shape” ([4] p. 87), correspond to predictions of the ontogenetic scaling hypothesis [4, 8]. Secondly, changes in adult shape can occur due to departure from the ancestral growth pathway: changes to the relationship between size and shape (on a bivariate plot of size and shape, changes in slope, intercept, or a combination of both). This instance may be inferred when ontogenetic variation among members of a group does not map onto a common ontogenetic trajectory. Generally it was thought that growth pathways are phylogenetically stable, and that the ontogenetic scaling hypothesis can explain most variation [18, 19]. However, growth pathways certainly can change; closely related taxa can show varying patterns of heterochrony [14, 20, 21], and ontogenetic divergence [22] and convergence [23]. Variation in growth patterns among related taxa show that selection can rapidly modify postnatal developmental pathways under some circumstances (e.g. [12–14, 24]).

Differences in ontogenetic patterns have often been associated with differences in ecology, in an interplay between selective forces and developmental processes. Evolutionary radiations consisting of many closely related species provide opportunities to examine how changes to an ancestral growth pathway may have contributed to morphological diversification within a particular clade. The Australian radiation of dragon lizards, the Amphibolurinae (Agamidae), includes iconic species such as the frill-neck lizard, bearded dragon, and thorny devil. They constitute a diverse component of Australia’s reptile fauna comprising around 108 species, and probably represent the descendants of a single continental colonisation from Southeast Asia approximately 30 million years ago (Ma) [25–27]. They diversified into a range of distinct morphotypes and ecological niches as the continent became increasingly arid [28, 29], and today they are particularly diverse in the arid zone [30, 31]. Among the most ecomorphologically relevant features of lizards is their head morphology (e.g. [32]); its role in supporting sensory structures, in food gathering, for social signalling and as a weapon, mean that it must be responsive to multiple selective pressures. Amphibolurinae includes some markedly varied and specialised skull shapes [33, 34], but apart from one recent limited study [35], there has been little examination of cranial growth patterns among different species.

This study aims to investigate whether the evolution of adult cranial shape diversity among 18 species of the Amphibolurinae is achieved through heterochrony alone, without changes to the ancestral growth pathway (as predicted by the ontogenetic scaling hypothesis), or through heterochrony and modification of these pathways. We use two-dimensional landmark based geometric morphometrics to characterise cranial shape, and compare the direction and magnitude of postnatal growth trajectories using a phenotypic trajectory analysis [36]. We also test for associations between ontogenetic patterns and life habit [37], and whether there is a phylogenetic signal in juvenile or adult skull shapes [38].

## Results

### Variation in cranial shape

**Fig. 1:**
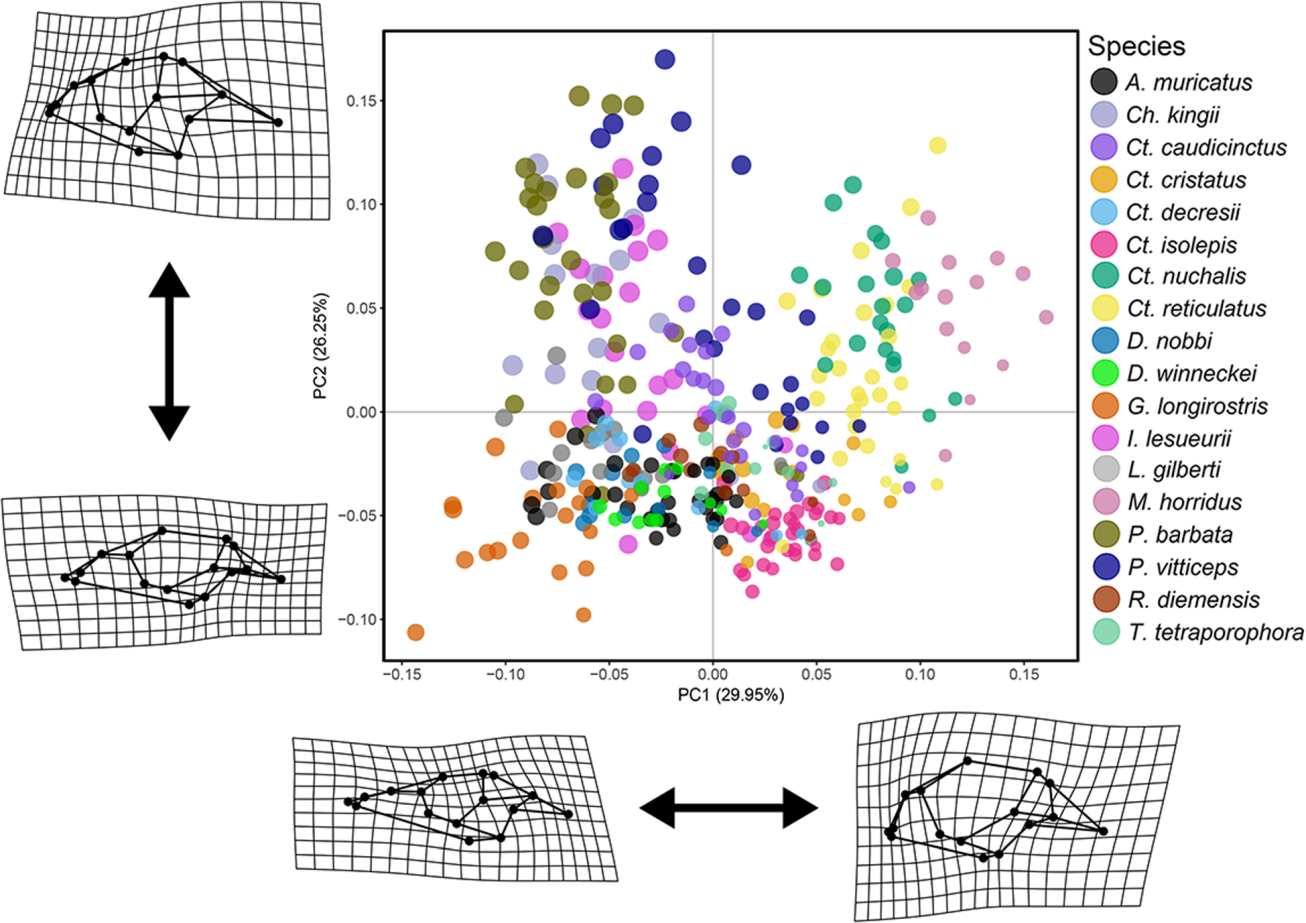
Cranial morphospace representing the two main axes of shape variation from a PCA of the Procrustes aligned landmark coordinates. Points are coloured according to species affiliation, and scaled according to centroid size. TPS deformation grids and wireframes represent shape changes between landmarks of the mean shape and minimum and maximum values of PC1 and PC2

A principal component analysis (PCA) characterising overall cranial shape across the sampled specimens shows that most of the smaller individuals have high PC1 values and low PC2 values (Fig. 1), which characterise skulls with relatively larger orbits and relatively shorter, smaller, and more slender post-orbit elements (jugal, postorbital, squamosal, parietal), and short blunt snouts. The other three quadrants of the morphospace are each associated with one of three major adult morphotypes. *Gowidon longirostris* is one example of an extreme morphotype (low PC1 values and low PC2 values) which has a relatively long and pointed snout, long maxillary facial process, a wide postorbital bar (jugal), small orbit, and an overall dorsoventrally shallow skull. *Chlamydosaurus kingii*, *Intellagama lesueurii*, *Pogona barbata* and *P. vitticeps* have relatively short snouts (compared to the most long snouted forms) and large, robust post-orbit elements (low PC1 values and high PC2 values). Interestingly, these four species are also those with the largest adult size. *Ch. kingii* and both species of *Pogona* have relatively short but pointed snouts, whereas *I. lesueurii* has a more rounded snout. The third extreme form of shape variation has a short and blunt snout, robust post-orbit elements, and a dorsoventrally deep overall skull profile, e.g. *Ctenophorus nuchalis*, *Ct. reticulatus* and *Moloch horridus*. For any particular species, the intermediate and adult ontogenetic stages occupy morphospace between the large-orbit form associated with smaller individuals (high PC1 values and low PC2 values), and one of these three broad morphotypes.

### Examining ontogenetic allometry

Morphological disparity (Procrustes variance) for Amphibolurinae was significantly (***P*** **= 0.001**) greater among adult crania for each species (mean of the three largest specimens, Procrustes variance = 0.0148) than among juvenile crania of each species (mean of the three smallest specimens, Procrustes variance = 0.0099). The disparity calculated for the smallest juvenile and largest adult representatives of each species show that different agamid species begin life with a similar cranial shape and later disperse towards more disparate adult forms.

**Fig. 2:**
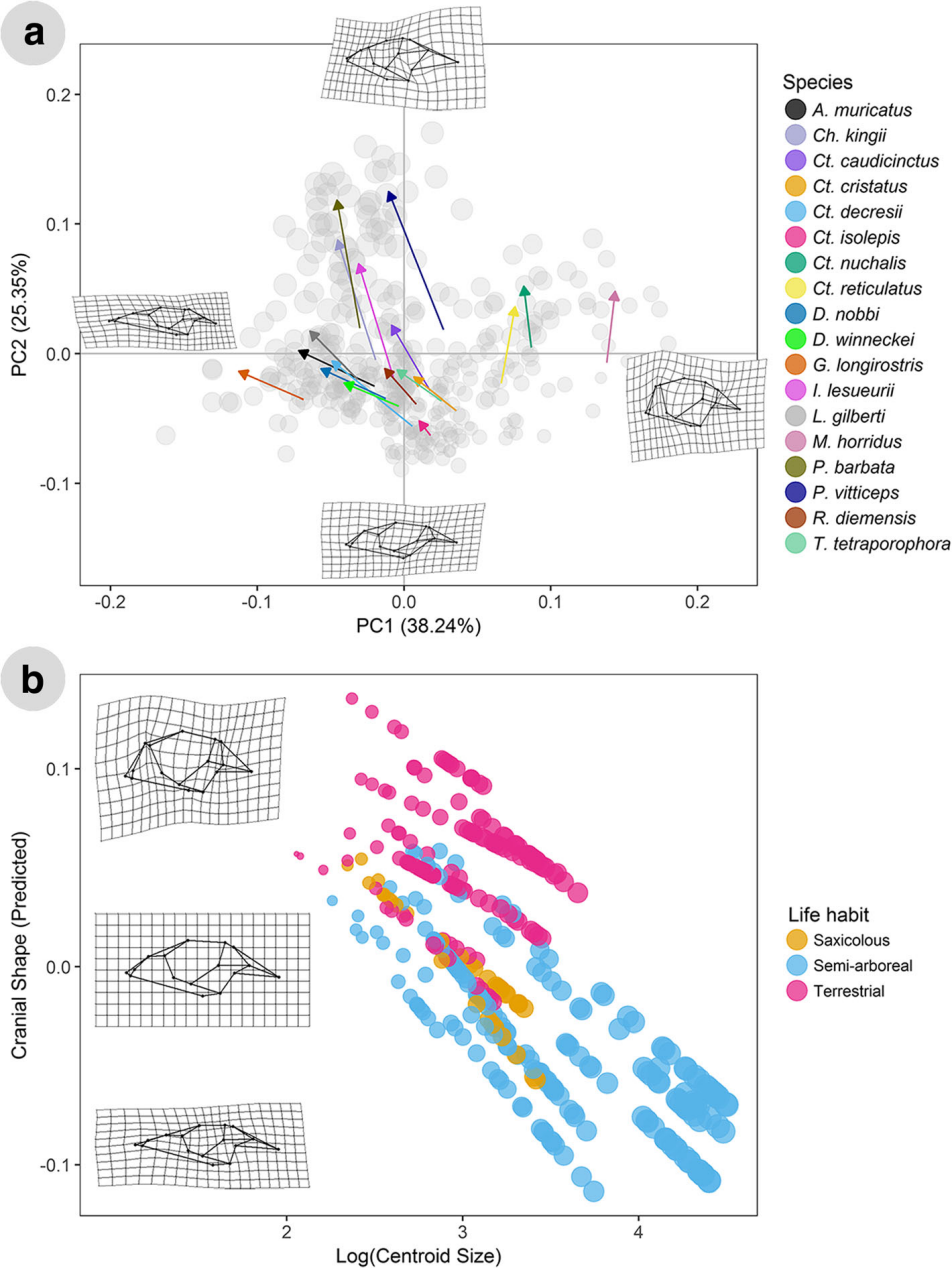
Ontogenetic allometric trajectories derived from the phenotypic allometric trajectory analysis (**a**) and the species ontogenetic allometric patterns identified by life habit (**b**). For both plots, the size of points for each specimen is scaled to centroid size. In a, specimens are plotted on a morphospace represented by PCs 1 and 2 on the x and y axes respectively. The arrows represent predicted trajectories for each species. Vectors represent ontogenetic trajectories of different species. The start of an arrow represents the mean juvenile shape and the end of the arrow represents the mean adult shape. The grey points represent the total variation within the sample. TPS deformation grids represent the shape change from the mean shape of the data set to the shape at the minimum and maximum values on that axis. In b, the x-axis represents log-transformed centroid size, and the y-axis represents the first principal component of the predicted values of multivariate regression of shape on size (as identified by MANCOVA). TPS deformations grids represent the shape change from the mean shape of the data set to the shape of the smallest and largest specimens in the data set

Tests for isometry in growth patterns for each species indicated that all species have significant allometric growth (lack of isometry). The variation detected in the shape data by a phenotypic trajectory analysis (PTA) revealed significant differences in growth trajectories: among directions (angles) of shape change, and also among magnitudes of shape change (Fig. 2a). For PC1, most species trajectories move from high values as juveniles, to low values as adults. For PC2, most species trajectories move from low values as juveniles, to high values as adults.

The direction of ontogenetic shape change varies between different species (Fig. 2a). Pairwise *P*-values for direction (angle) differences in the PTA are reported in Additional file 1: Table S2. Out of 153 possible pairs of species, 74 shared a common slope, while the remaining 79 pairs had significantly different directions of ontogenetic shape change. The pairwise results show that there are several cases where members belonging to the same clade or genus have different directions of ontogenetic shape change (e.g. *Ctenophorus isolepis* and *Ct. nuchalis*). *M. horridus* has a significantly different direction of shape change than all other sampled species. *I. lesueurii*, *Ch. kingii*, *P. barbata*, and *P. vitticeps* have the largest adult size, and all have similar directions of ontogenetic shape change. The species with the smallest adult sizes are *Ct. isolepis, Diporiphora winneckei, Rankinia diemensis, Tympanocryptis tetraporophora,* and *M. horridus,* and they also have mostly similar directions of ontogenetic shape change, apart from *M. horridus*.

There are significant differences in the magnitudes of shape change between different species (Fig. 2a). Out of 153 possible pairs of species, 90 had a similar magnitude of shape change, while the remaining 63 pairs had significantly different magnitudes of shape change (Additional file 1: Table S3). Species in this study with a larger adult size (*Ch. kingii, I. lesueurii, P. barbata, P. vitticeps*) have greater magnitudes of ontogenetic shape changes than other sampled agamids. *Ct. isolepis* shows the smallest magnitude of shape change compared with all other sampled species. *Ct. cristatus, Ct. decresii*, *Ct. nuchalis and Ct. reticulatus* all have relatively small magnitudes of shape change. In *Ctenophorus*, only three significant pairwise differences in magnitude were detected (both involving *Ct. isolepis*). Within the sister clade to *Ctenophorus* we detected 21 pairwise differences, mostly involving *Ch. kingii* and the species of *Pogona,* which show the largest magnitude of shape change of any species (Additional file 1: Table S3).

Cranial shape is influenced by cranial size, life habit and the interactions of the two (MANCOVA, size F_(1, 361)_ = 128.35, ***P*** **= 0.001**; habit F_(3, 361)_ = 39.83, ***P*** **= 0.001**; size*habit F_(3, 361)_ = 5.24, ***P*** **= 0.001**). Thus, there is significant allometry in cranial shape, and the allometric slopes differ among life habit categories (see also Additional file 2: Table S4). The differences in ontogenetic allometric patterns (log transformed centroid size vs. predicted cranial shape) between species with different life habits is evident in Fig. 2b. Pairwise comparisons of allometric trajectories revealed that all life habit groups have significantly different slopes (direction of shape change) from one another, except for the semi-arboreal and saxicolous groups. There were also significant pairwise differences in trajectory length (amount of shape change) detected in pairwise comparisons for all three life habit groups (see Additional file 2: Table S5 for *P*-values for pairwise angle and length differences for life habit groups).

### Phylogenetic signal

**Fig. 3:**
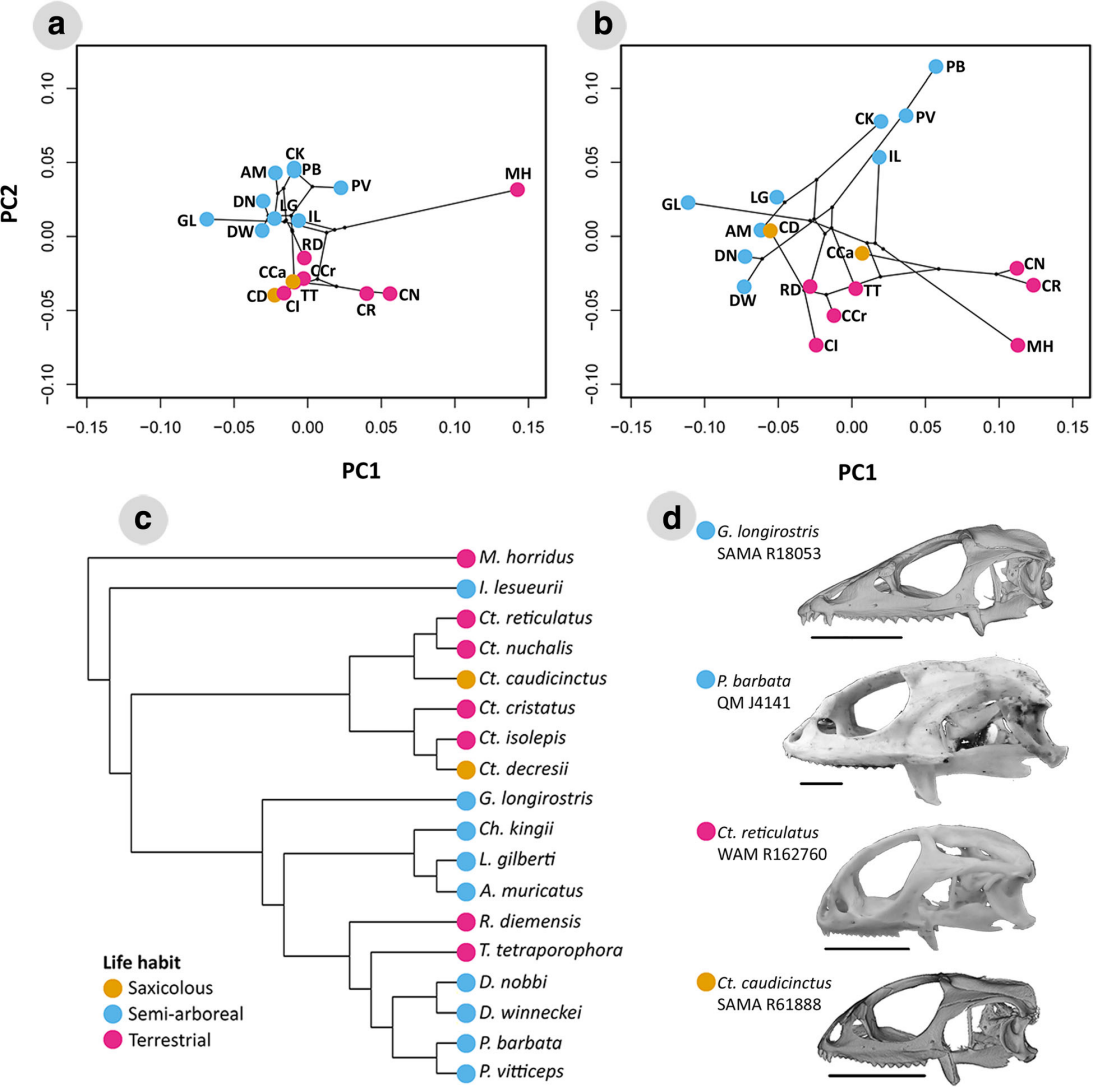
Phylomorphospaces (PC1 versus PC2) for smallest juvenile (**a**) and largest adult shapes (**b**). Abbreviations are as follows: *AM A. muricatus*, *CH Ch. kingii*, *CCa Ct. caudicinctus*, *CCr Ct. cristatus*, *CD Ct. decresii*, *CI Ct. isolepis*, *CN Ct. nuchalis*, *CR Ct. reticulatus*, *DN D. nobbi*, *DW D. winneckei*, *GL G. longirostris*, *IL I. lesueurii*, *LG L. gilberti*, *MH M. horridus*, *PB P. barbata*, *PV P. vitticeps*, *RD R. diemensis*, *TT T. tetraporophora*. **c** shows the inferred phylogenetic tree of relationships between agamids used in this study. All points are coloured according to life habit category. **d** shows examples of adult skulls that represent extreme variation in shape and different life habits. Scale bar = 10 mm

Tests for phylogenetic signal (relative to what is expected under a Brownian motion model of evolution) in cranial shape of the smallest juveniles and the largest adults revealed that both juveniles and adults show significant phylogenetic signal in their shape (juvenile: K_mult_ = 0.44, ***P*** **= 0.001**; adult: K_mult_ = 0.44, ***P*** **= 0.001**). The amount of phylogenetic signal is less than 1, indicating that species resemble each other less than is expected under Brownian motion. This is reflected in the dispersion of the species in the two cranial phylomorphospaces (Fig. 3), where there are many overlapping branches and where closely-related species are not adjacent in morphospace. The K_mult_ estimates are the same for both the juvenile and adult shape data, despite the different dispersion patterns in the two phylomorphospaces. Conversely, there was no significant phylogenetic signal detected in cranial size (juveniles: *K* = 0.31, *P* = 0.130; adults, *K* = 0.36, *P* = 0.063).

## Discussion

The amphibolurine post-natal growth pathway is evolutionarily flexible and has played a major role in producing great morphological disparity in adult cranial shape. Shape variation in both juveniles and adults had some phylogenetic signal, indicating that inheritance plays some role in structuring the morphological and ontogenetic variation we observe. However, different ontogenetic patterns are significantly associated with different life habits, suggesting that a significant amount of the variation in adult cranial shape is adaptive. Overall, it appears that environmental pressures often overwrite evolutionary history to influence ontogenetic patterns of skull shape in this radiation of lizards.

Most of the disparity in the adult cranial form of the sampled amphibolurines develops during post-hatching ontogeny. For just under half of the sampled species, we observe shape changes along a shared allometric slope, with indicates paedomorphy as a particular heterochronic pattern in a number of species of *Ctenophorus*. This finding is consistent with the ontogenetic scaling hypothesis (changes in the time or rate of development along the growth pathway). However, heterochrony is insufficient to explain the entirety of high morphological diversity of the amphibolurines. More often than not, we observe differences in both direction and magnitude of growth pathways between pairs of species, which suggests ontogenetic divergence has been a major factor in the evolution of the disparity seen in adult amphibolurine lizards.

The extent to which ontogenies are conserved during evolution has been a continued topic of controversy that has influenced the development of phylogenetic methods [8, 10, 39, 40]; this study adds to the growing amount of evidence that morphological ontogenies are as prone to selection and evolutionary change as other aspects of morphology. It has been suggested that changes in the direction of allometric slopes are rare, since they represent costly alterations to growth dynamics [7–9], but our study adds to the growing literature that shows such changes are quite common [13, 14, 41]. The importance of evolving ontogenies in generating morphological diversity in amphibolurines resembles what has been shown in other reptiles [16], other vertebrates [42, 43], and even in plants [44]. In some cases, developmental pathways do represent an evolutionary constraint, canalising the phenotypic variation of species into particular portions of morphospace, as has been reported in lacertid lizards [45]. It seems that ontogenies are more flexible than previously thought, and can allow morphology to explore previously unoccupied areas of morphospace.

In both juvenile and adult skull shapes, phylogenetic affinity is less strongly correlated with shape than are associations with ecological groups. Although we find strong associations with ecological life habit, this does not preclude the existence of other many important factors, such as diet or patterns of interspecific selection. The weak phylogenetic signal we observe agrees with other studies on the skulls of reptiles [21, 46], mammals [47, 48], and fish [49], that have identified greater associations between morphology and diet [12, 47], feeding habits [49, 50], habitat [24, 51], and environmental factors [49, 52], than with phylogeny. In contrast, some studies confirm a strong phylogenetic signal in morphological variation, such that ecological correlations are not evident [51, 53, 54] or have minimal effect [55]. Although adaptive factors and phylogeny both undoubtedly play a role in shaping morphological diversity, the extent of this role evidently differs amongst clades and should be assessed on a case-by-case basis.

Our findings add to the growing body of evidence [5, 12, 36, 37, 56] that highlights the importance of using a rigorous quantitative framework to investigate the underlying basis of phenotypic variation. We have yet to investigate the influence of sexual dimorphism on amphibolurine lizards because of the lack of sex information for most of the museum specimens studied. Sexual size dimorphism has been recorded for at least one species in this study (see [57, 58]), and therefore we cannot discount the possibility that it has an influence on skull shape, considering the strong allometric effects observed in these lizards. Therefore, future studies are encouraged to investigate the effect of sexual dimorphism on morphological variation in this clade. Furthermore, the influence on growth of diet and feeding habits on skull shape are yet to be studied in this group but are required to fully understand the evolutionary patterns we have observed. Our study thus serves as foundation for further studies to examine hypotheses about these factors and more.

## Conclusions

Diversity in cranial shape of amphibolurine lizards seems to be the result of a combination of heterochrony and changes in growth patterns, which are related to phylogenetic affinity and adaptive evolution. The expectation of a conserved phylogenetic pattern, as predicted by the ontogenetic scaling hypothesis, does not fully explain the variation in skull shapes, and we find compelling evidence that there is an adaptive basis for much of the variation in ontogenetic allometry that we observe. Our study emphasises the power of growth pathways for facilitating the morphological variation that is characteristic of large and speciose evolutionary radiations. It also underlines the importance of using quantitative multivariate analyses to properly appreciate the role of developmental processes in shaping phenotypic diversity across species.

## Methods

### Study specimens

Material comprised 2D lateral view images of 361 specimens representing 18 different species of amphibolurine lizards (Table 1). The species were chosen to optimise taxonomic breadth, skull shape diversity, and size, but limited to species where the sample size was 10 specimens or more and included both juveniles and adults. We collected data from skeletal specimens from several institutions including South Australian Museum, University of Texas at Austin, Western Australian Museum, Field Museum of Natural History, Queensland Museum, University of Adelaide, and Melbourne Museum.

**Table 1:** Species studied

Species	n	Average adult skull length (mm)	Life habit
*Ctenophorus caudicinctus*	26	22.43	Saxicolous
*Ctenophorus cristatus*	15	24.39	Terrestrial
*Ctenophorus decresii*	10	23.51	Saxicolous
*Ctenophorus isolepis*	30	17.72	Terrestrial
*Ctenophorus nuchalis*	21	29.07	Terrestrial
*Ctenophorus reticulatus*	29	26.05	Terrestrial
*Amphibolurus muricatus*	34	28.72	Semi-arboreal
*Chlamydosaurus kingii*	17	75.97	Semi-arboreal
*Diporiphora nobbi*	12	23.22	Semi-arboreal
*Diporiphora winneckei*	12	13.37	Semi-arboreal
*Gowidon longirostris*	20	32.65	Semi-arboreal
*Lophognathus gilberti*	16	31.80	Semi-arboreal
*Pogona barbata*	29	64.83	Semi-arboreal
*Pogona vitticeps*	29	60.46	Semi-arboreal
*Rankinia diemensis*	12	18.97	Terrestrial
*Tympanocryptis tetraporophora*	11	15.93	Terrestrial
*Intellagama lesueurii*	22	70.96	Semi-arboreal
*Moloch horridus*	15	16.31	Terrestrial

Sample sizes were dependent on availability from collections. Average skull length was calculated using the basal skull length of the largest three individuals of each species. Life habit categories were based on records in Wilson and Swan [65] and Cogger [66]

### Imaging

The left side of the cranium was studied from 2D images. Each skeletal specimen was oriented using dry sand (black in colour for contrast) and photographed using an Olympus TG-4 camera mounted on a large flexible tripod, with a ruler set to the sagittal (midline) axis of the skull as a reference for scale. We also used 2D images of 3D rendered surface models generated from micro-Computed Tomography (CT) reconstructions of specimens in alcohol from South Australian Museum. These specimens had been micro CT scanned at either ~ 18 or ~ 9 μm resolution (depending on the size of the specimen) using a Skyscan 1076 (Bruker micro-CT) at Adelaide Microscopy. Each CT scan was reconstructed using the NRecon software interface [59]. We used Avizo v 9.0 [60] to digitally segment the cranium, threshold non-bone components from the scan, and render a surface model, which was then oriented laterally to capture a 2D image within Avizo, with a scale bar.

### Landmarks and shape analysis

Lateral cranial shape was characterised using 2D landmark-based geometric morphometrics. Landmarks were digitised on the images of the crania using tpsDig v. 2.21 [40]. We set the scale for each specimen using scale bars present in the digital images, and digitised 16 single point landmarks (see Additional file 3: Figure S1), that represented equivalent points on bones at suture junctions, boundaries, and extremes of curvature on structures (see Additional file 4: Table S1, for landmark definitions). All subsequent analyses were performed using a routine written for the R statistical framework v 3.4.0. The raw 2D landmark coordinates were subjected to a generalised Procrustes alignment (GPA) using the R package *geomorph* [61]. This effectively removed differences in size, position, and orientation, leaving only shape variation [62]. The resulting Procrustes aligned shape coordinates were used as shape variables in subsequent analyses. Centroid size (the square root of the sum of the square distances of each landmark before GPA) was used as a proxy for body size. We were unable to use snout-vent length measurements because this data was not available for most of the skeletal specimens used.

### Visualising shape variation

We performed a principal component analysis (PCA) on the Procrustes aligned shape coordinates to visualise the variation among sets of landmarks in the data set. To interpret the shape differences described by the major axes of variation identified by the PCA, we plotted a morphospace (PC1 versus PC2) with points identified by species and scaled to represent centroid size. To visualise the shape variation associated with the major axes of variation, we used thin-plate spline deformation grids [63], produced using the “plotRefToTarget” function in *geomorph*, and a wireframe representation of the skull, to represent shape differences between corresponding landmarks of the mean shape and minimum and maximum values for PC1 and PC2.

### Examining allometry

To examine whether the morphological disparity in cranial shape among species differs between juveniles and adults, we quantified the disparity for two separate groups: the smallest three juveniles of each species (start of growth trajectory); and the largest three adults of each species (end of growth trajectory). We calculated morphological disparity using the “morphol.disparity” function in *geomorph*, which estimates Procrustes variance while accounting for group size, and uses absolute differences in variances to test for pairwise differences in morphological disparity between groups. The statistical significance between the juvenile and adult groups was assessed using a randomised residual permutation test with 1000 iterations.

We determined whether any species displayed isometric growth (no change in shape with a change in size), by fitting individual regression of log-transformed size on shape for each species, using the “procD.lm*”* function from the R package *geomorph*, which assesses significance via distributions generated with resampling permutations (we used 1000 iterations). If the association between size and shape for a particular species was significant, this result indicated that there was an ontogenetic allometric (and not isometric) pattern present for that particular species.

To test whether ontogenetic trajectories differ among species we conducted a phenotypic trajectory analysis (PTA) [64] on the shape coordinates using the “trajectory.analysis” function in *geomorph*. This procedure quantifies different attributes of a shape change trajectory between two or more points, in this case we measure the attributes of shape change between two groups: juveniles and adults. To circumvent issues with estimating nearness to adulthood in a clade with such broad variation in adult body sizes, we were able to categorise each specimen as either a juvenile or adult based on the number of acrodont teeth they had. If a specimen had more than 80% of the maximum number of acrodont teeth observed for that species, they were categorised as an adult. In some cases we altered the categorisation if a specimen was missing enough teeth to hinder obtaining a count, and were able to categorise these as either juveniles or adults based on the centroid sizes observed for other specimens of that particular species. We used species as groups, and juveniles and adults as the trajectory points. This analysis involved pairwise comparisons of two different attributes: the magnitudes of the trajectories among species, and also directions of the trajectories among species. Attribute differences were evaluated from sampling distributions generated from 1000 random permutations (based on a null model that lacked coefficients for a species-transect interaction) [36]. To visualise the ontogenetic phenotypic trajectories, we plotted the first two PCs of shape variation with arrows representing vectors of shape change (where the start of the arrows represents mean juvenile shapes and the end of the arrows represents mean adult shapes) and used thin-plate spline deformation grids to visualise shape change.

We tested for differences in ontogenetic allometric patterns of skull shape among ecological life habit groups by running a multivariate analysis of covariance (MANCOVA) model using the *geomorph* function “procD.allometry”, with log transformed centroid size, life habit, and their interaction as model effects. Life habit was split into three categories (see Table 1), based on information available in Wilson and Swan [65] and Cogger [66]. Statistical significance was evaluated using Goodall’s [67] F-ratio and a randomised residual permutation procedure using 1000 iterations [37]. If the interaction terms were significant, this indicated that the allometric trajectories differed among life habit groups. We identified which life habits groups differed from each other, using the “advanced.procD.lm” function in *geomorph*. These tests identified which life habit groups significantly differed in allometric slope from each other, via pairwise assessments of the similarity in slopes and intercepts through 1000 randomised residual permutations. To visualise ontogenetic allometric trajectories of species with different life habits, we plotted the predicted shape scores (from a multivariate regression of shape ~ log (size) *species), on log transformed centroid size, and identified points by life habit.

We inferred an evolutionary tree using Hugall et al., Melville et al., and Pyron et al. [25, 29, 68], and used this tree to estimate phylogenetic signal present in shape and size of the smallest juveniles and adults, relative to what is expected for the inferred phylogeny under a Brownian motion model of evolution. We used the mean shape of the smallest three individuals (by centroid size) for each species to estimate phylogenetic signal in juvenile shapes, and the mean of the largest three individuals (by centroid size) of each species to estimate phylogenetic signal in adult shapes. To estimate phylogenetic signal we calculated K_mult_ [38], which is a generalisation of Blomberg’s K-statistic appropriate for high-dimensional and multivariate data [69]. We determined statistical significance of K_mult_ using phylogenetic permutation with 1000 iterations, which is calculated by permuting the multivariate shape data of the specimens among all tips of the phylogenetic tree. This was done using the “physignal” function in *geomorph*. To visualise how shape variation among species is associated with phylogeny we carried out separate PCAs on the landmark data for the mean shape of the three smallest juveniles and mean shape of the three largest adults, and used the phylomorphospace approach to project a phylogeny into the juvenile and adult PC biplots (with internal nodes estimated using maximum likelihood), implemented with the function “phylomorphospace” in the R package *phytools* [70].

## Additional files

Additional file 1: **Table S1.** Pairwise results for angles in phenotypic trajectory analysis. Upper triangle shows pairwise *p*-values, while lower triangle shows pairwise angle differences (red highlighted cells indicate *p* < 0.05). **Table S2.** Pairwise results for magnitude in phenotypic trajectory analysis. Upper triangle shows pairwise *p*-values, while lower triangle shows pairwise magnitude differences (red highlighted cells indicate *p* < 0.05). (XLSX 19 kb)

Additional file 2: **Table S3.** Examining allometry: MANCOVAs of cranial shape predicted by size and life habit (shape ~ log(size)*habit). **Table S4.** Examining allometry of life habit groups: pairwise angle and length differences. Upper triangle = *p*-values. Lower triangle = angles. (DOCX 15 kb)

Additional file 3: **Figure S1.** Images of *A. muricatus* with landmark numbers which correspond to landmark definitions found in Additional file 4. (PPTX 2107 kb)

Additional file 4: **Table S5.** Landmark definitions used in geometric morphometric analysis (follows Evans 2008 nomenclature of skeletal elements). (DOCX 12 kb)

## Abbreviations

2D: two-dimensional
3D: three-dimensional
AM: *A. muricatus*
CCA: *Ct. caudicinctus*
CCR: *Ct. cristatus*
CD: *Ct. decresii*
CH: *Ch. kingii*
CI: *Ct. isolepis*
CN: *Ct. nuchalis*
CR: *Ct. reticulatus*
CT: computed tomography
DN: *D. nobbi*
DW: *D. winneckei*
GL: *G. longirostris*
GPA: Generalised Procrustes alignment
IL: *I. lesueurii*
LG: *L. gilberti*
MANCOVA: Multivariate analysis of covariance
MH: *M. horridus*
Ma: million years ago
PB: *P. barbata*
PC: Principal component
PCA: Principal component analysis
PTA: Phenotypic trajectory analysis
PV: *P. vitticeps*
RD: *R. diemensis*
TT: *T. tetraporophora*
