# Supplementary material for: Changes in ontogenetic patterns facilitate diversification in skull shape of Australian agamid lizards
Source: BMC Evol Biol. 2019 Jan 8;19:7. doi: 10.1186/s12862-018-1335-6 (PMC6325775; doi:10.1186/s12862-018-1335-6)
Supplement: Supplementary file 2 — Table S3. Examining allometry: MANCOVAs of cranial shape predicted by size and life habit (shape ~ log(size)*habit). Table S4. Examining allometry of life habit groups: pairwise angle and length differences. Upper triangle = p-values. Lower triangle = angles. (DOCX 15 kb) [file 12862_2018_1335_MOESM2_ESM.docx]

**Table S3.** Examining allometry: MANCOVAs of cranial shape by size and life habit (Y ~ size*habit).

|  | Df | SS | MS | Rsq | F | Z | P |
| --- | --- | --- | --- | --- | --- | --- | --- |
| Life habit allometry |  |  |  |  |  |  |  |
| log (size) | 1 | 0.9360 | 0.93502 | 0.224809 | 128.3495 | 8.6221 | **0.001** |
| habit | 2 | 0.5809 | 0.29044 | 0.139513 | 39.8258 | 9.7491 | **0.001** |
| Log(size):habit | 2 | 0.0578 | 0.02890 | 0.013883 | 3.9631 | 5.2379 | **0.001** |
| residuals | 355 | 2.5889 | 0.00729 |  |  |  |  |
| total | 360 | 4.1636 |  |  |  |  |  |

**Table S4.** Examining allometry of life habit groups: pairwise angle and length differences. Top triangle = p-values. Bottom triangle = angles.

|  | Saxicolous | Semi-arboreal | Terrestrial |
| --- | --- | --- | --- |
| **Angle** |  |  |  |
| Saxicolous | - | 0.114 | **0.005** |
| Semi-arboreal | 34.07 | - | **0.001** |
| Terrestrial | 53.58 | 36.16 | - |
| **Length** |  |  |  |
| Saxicolous | - | **0.013** | **0.001** |
| Semi-arboreal | 0.013405 | - | **0.015** |
| Terrestrial | 0.001075 | 0.01448 | - |
