# Supplementary material for: Changes in ontogenetic patterns facilitate diversification in skull shape of Australian agamid lizards
Source: BMC Evol Biol. 2019 Jan 8;19:7. doi: 10.1186/s12862-018-1335-6 (PMC6325775; doi:10.1186/s12862-018-1335-6)
Supplement: Supplementary file 3 — Figure S1. Images of A. muricatus with landmark numbers which correspond to landmark definitions found in Additional file 4. (PPTX 2107 kb) [file 12862_2018_1335_MOESM3_ESM.pptx]

## Slide 1
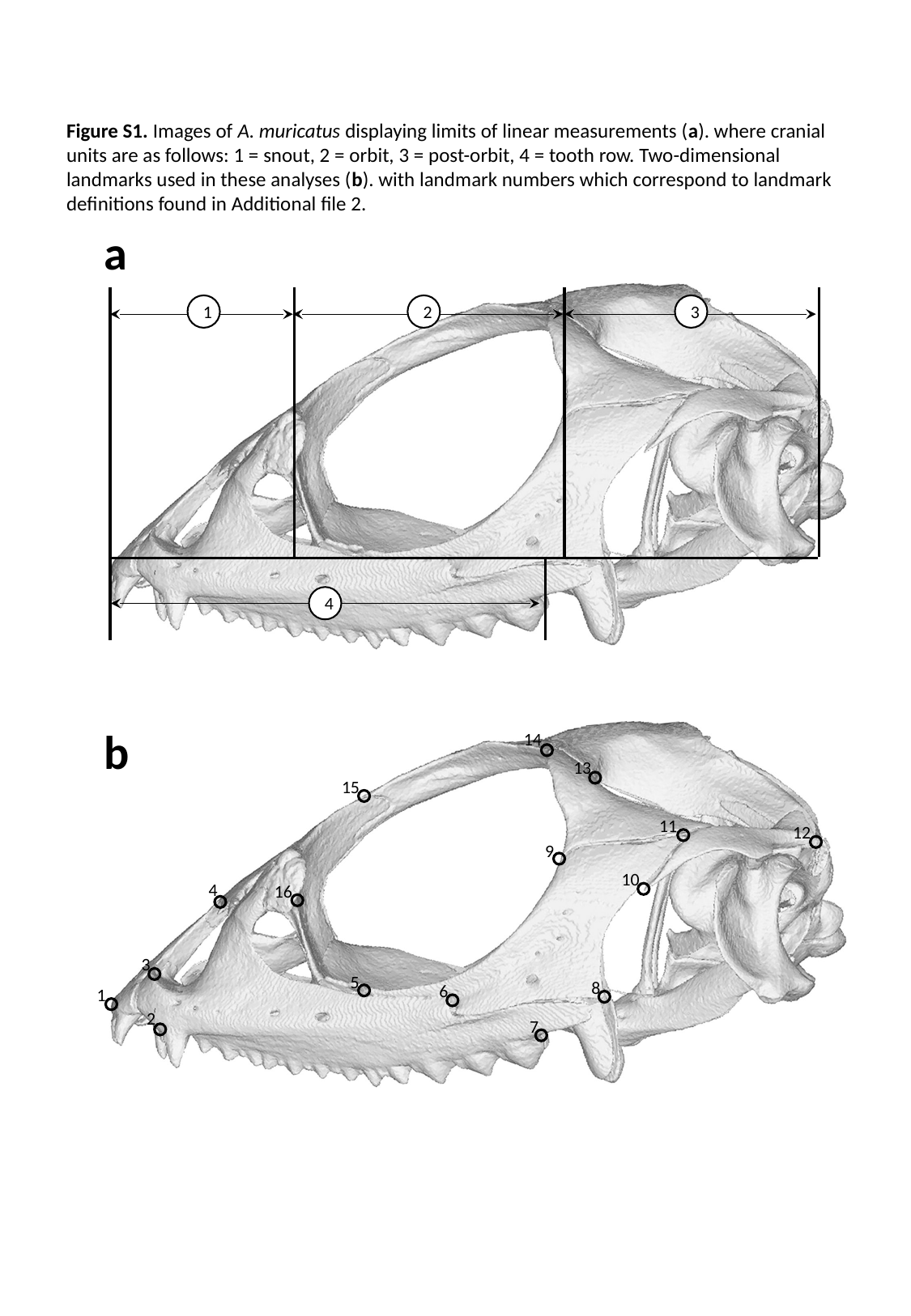

Figure S1. Images of A. muricatus displaying limits of linear measurements (a). where cranial units are as follows: 1 = snout, 2 = orbit, 3 = post-orbit, 4 = tooth row. Two-dimensional landmarks used in these analyses (b). with landmark numbers which correspond to landmark definitions found in Additional file 2.
a
1
2
3
4
b
14
13
15
11
12
9
10
4
16
3
5
8
6
1
2
7
