# Supplementary material for: Changes in ontogenetic patterns facilitate diversification in skull shape of Australian agamid lizards
Source: BMC Evol Biol. 2019 Jan 8;19:7. doi: 10.1186/s12862-018-1335-6 (PMC6325775; doi:10.1186/s12862-018-1335-6)
Supplement: Supplementary file 4 — Table S5. Landmark definitions used in geometric morphometric analysis (follows Evans 2008 nomenclature of skeletal elements). (DOCX 12 kb) [file 12862_2018_1335_MOESM4_ESM.docx]

| Number | Description |
| --- | --- |
| 1 | Anterior limit of the snout |
| 2 | Anterior limit of the base of the most anterior maxillary tooth |
| 3 | Most dorsal point of the anterior process of the maxilla |
| 4 | Dorsal limit of the nasal opening |
| 5 | Anterior limit of the jugal |
| 6 | Posterior limit of the maxillary posterior dorsal process |
| 7 | Posterior limit of the base of the most posterior acrodont tooth |
| 8 | Most postero-ventral point of the jugal |
| 9 | Most antero-ventral point of the postorbital |
| 10 | Anterior limit of the squamosal |
| 11 | Posterior limit of the jugal |
| 12 | Posterior limit of the squamosal |
| 13 | Most posterior point of the postorbital-parietal suture |
| 14 | Most anterior point of the postorbital-parietal suture |
| 15 | Most dorsal point of the prefrontal-frontal suture |
| 16 | Anterior limit of the orbital opening |

**Table S5**. Landmark definitions used in geometric morphometric analysis (see Evans 2008 for nomenclature of skeletal elements).
